# Review on the Antimicrobial Resistance of Pathogens from Tracheal and Endotracheal Aspirates of Patients with Clinical Manifestations of Pneumonia in Bacolod City in 2013

**DOI:** 10.1155/2015/942509

**Published:** 2015-02-03

**Authors:** Alain C. Juayang, Dominador G. Maestral, Gemma B. de los Reyes, Michael Angelo D. Acosido, Christine T. Gallega

**Affiliations:** Medical Technology Program, College of Arts, Sciences and Education, Colegio San Agustin-Bacolod, 6100 Bacolod City, Philippines

## Abstract

Microbiological content specifically bacterial and fungal etiologies from tracheal aspirates in a tertiary hospital in Bacolod City was reviewed for baseline information. A total of 130 tracheal aspirates were subjected for culture to isolate and identify the pathogen and determine their susceptibilities to various antibiotics. Productions of certain enzymes responsible for antibiotic resistance like ESBL (Extended Spectrum Beta-Lactamase), metallo-*β*-lactamase, and carbapenemase were also studied. Out of 130 specimens, 69.23% were found to be positive for the presence of microorganisms. Most infections were from male patients aging 60 years and above, confined at the Intensive Care Units (ICU). *Pseudomonas aeruginosa* and *Klebsiella pneumoniae* were found to be the most frequent bacterial isolates and non-*Candida albicans* for fungal isolates, respectively. Among the various antibiotics tested, most isolates were found to be resistant to third generation cephalosporins and penicillins, but susceptible to aminoglycoside Amikacin. On the other hand, production of ESBL and carbapenemase was found to be common among members of Enterobacteriaceae especially *K. pneumoniae*.

## 1. Introduction

Endotracheal specimens or tracheal aspirates are usually done quantitatively in culture in order to determine the presence of pneumonia in ventilated patients admitted in most hospitals rather than bronchoalveolar lavage (BAL) or protected specimen brush (PSB) and are considered as reliable alternative compared to the latter specimens [1–3]. These specimens are often used in the diagnosis of pneumonia, including ventilator acquired pneumonia. In the hospital, tracheal aspiration for the diagnosis of nosocomial infection is also common in patients admitted in the ICU [4–6].

Several organisms are accounted for causing pneumonia. Common causative agents are that of* Pseudomonas* species,* Acinetobacter* species,* Staphylococcus aureus*, and Enterobacteriaceae including the endogenous bacteria [7–11]. The common problem however as noted in other studies is that bacterial pathogens from tracheal aspirates tend to be of a multidrug-resistant type [8–10]. Antibiotic treatment using piperacillin-tazobactam has shown efficacy in lowering hospital mortality rate [12], so as with fluoroquinolones, Amikacin, and carbapenems [8].

Although many foreign studies had investigated the microbiological content of tracheal and endotracheal aspirates, it is also worthwhile that locally gathered data will be given the same importance [13, 14]. These lead to the aim of the study to determine and enumerate the presence of pathogens and its antibiotic susceptibility pattern in endotracheal and tracheal aspirates from a tertiary hospital in Bacolod City, Philippines, for the year 2013 for baseline information.

## 2. Materials and Methods

### 2.1. Inclusion and Exclusion Criteria

This study was conducted from January 2013 to December 2013 in a Tertiary Hospital in Bacolod City, Philippines, and was patterned on the study of Khosravi et al. in 2013 [10]. Samples were derived from both admitted and outpatients who underwent intubation or tracheostomy procedure due to impaired respiratory or pulmonary functions like those who suffered severe COPD, acute asthma attack, and upper airway obstruction, those who fell in the GCS 7 and below, and so forth having manifestations of pneumonia as assessed by their attending physicians. These patients were then grouped according to whether they were outpatients, non-ICU patients, or ICU patients. These groups were then categorized according to age and gender. Specimens were collected by the attending physicians and were sent to the laboratory for microbial analyses. Samples with pus cells of more than 25/LPF were included in the study, whereas those with less than 25/LPF pus cells were excluded [3]. The study also considered microbial growth of 10^4^ CFU/mL or more for diagnostic threshold [2, 3].

### 2.2. Isolation and Identification of Pathogens

Submitted samples were then inoculated on Sheep's Blood Agar, Mac Conkey, and Chocolate Agar for routine bacterial and yeast isolation. Traditional and automated methods were employed in identifying the organisms based on their reaction in biochemical tests using both schemes in identifying species of Enterobacteriaceae and* Pseudomonas aeruginosa* [15]. Identification of fungal isolates was based on the study of Ogba et al. [16].

### 2.3. Antimicrobial Susceptibility Testing

Antimicrobial susceptibility testing was performed by Kirby-Bauer disc diffusion method strictly adhering to the standards stipulated in the M02-A11 [17] and M100-S23 of CLSI [18]. Inoculums were prepared using direct colony suspension and adjusted to that of 0.5 McFarland Standard solutions. A sterile swab was then immersed into the inoculum and swabbed on Mueller Hinton Agar II (MH) plates and allowed to dry for 5–15 minutes. The antibiotic discs were then placed 2 centimeters apart from each other. The plates were then incubated at 35–37°C for 18 to 24 hours and zone of inhibitions was measured with the use of Vernier caliper. The results were sequentially interpreted based on the guidelines stipulated in the M100-S23 standards [18].

Antibiotics that were used include Amikacin (30 *μ*g), Ampicillin-Sulbactam (10/10 *μ*g), Aztreonam (30 *μ*g), Co-Amoxiclav (20/10 *μ*g), Cefepime (30 *μ*g), Cefoxitin (30 *μ*g), Cefotaxime (30 *μ*g), Ceftriaxone (30 *μ*g), Ceftazidime (30 *μ*g), Cefuroxime (30 *μ*g), Chloramphenicol (30 *μ*g), Sulfamethoxazole-Trimethoprim (1.25/23.75 *μ*g), Ertapenem (10 *μ*g), Gentamycin (10 *μ*g), Imipenem (10 *μ*g), Meropenem (10 *μ*g), Tazo.Piperacillin (100/10 *μ*g), Tetracycline (30 *μ*g), Tobramycin (10 *μ*g), and Fluconazole (20 *μ*g). The data were encoded and analyzed using the WHONET version 5.6 downloaded from WHO website.

### 2.4. Detection of Extended Spectrum Beta-Lactamase

Screening and confirmatory tests for Extended Spectrum Beta-Lactamase (ESBL) were determined using the antibiotic discs Ceftazidime (30 *μ*g), Aztreonam (30 *μ*g), Cefotaxime (30 *μ*g), Ceftriaxone (30 *μ*g), Cefotaxime-clavulanic acid (30/10 *μ*g), and Ceftazidime-clavulanic acid (30/10 *μ*g) as mentioned in the M100-S23 [18]. For screening of ESBL, any isolate with a zone on Ceftazidime of ≤22 mm, Aztreonam—≤27 mm, Cefotaxime—≤27 mm, and Ceftriaxone <25 mm may indicate enzyme production. Isolates that fell in the aforementioned screening criteria were then phenotypically confirmed using disc potentiation assay utilizing Ceftazidime, Cefotaxime, Cefotaxime-clavulanate discs, and Ceftazidime-clavulanate discs. A ≥5 mm increase in zone diameter of either antimicrobial agent tested in combination with clavulanate versus the zone diameter of the agent when tested alone confers ESBL.

### 2.5. Detection of Metallo-Beta-Lactamase and Carbapenemase Production among Isolates

Determination of metallo-*β*-lactamase and carbapenemase was done in* Pseudomonas* and* Acinetobacter* isolates that were found to be resistant to the tested carbapenem using the Imipenem-EDTA synergistic test as mentioned in the study of Pitout et al. [19]. Modified Hodge Test was also used to the isolates of Enterobacteriaceae that were found to be resistant to any of the carbapenems based on the guidelines of CLSI [18].

#### 2.5.1. Combined Disk Test Using Imipenem and EDTA

This assay was based on the method mentioned in the study of Pitout et al. [19], wherein a 0.5 McFarland standardized inoculum of the test isolate was inoculated onto a MH agar. Two 10 *μ*g imipenem discs were initially placed onto the agar and 10 *μ*L of 930 *μ*g of EDTA solution is added to one of the imipenem disc. An isolate with a ≥7 mm difference in the zone of inhibition between Imipenem-EDTA disc and Imipenem disc is considered as an MBL-producer.

#### 2.5.2. Modified Hodge Test

Confirmatory Test for Suspected Carbapenemase Production or the Modified Hodge Test was done by preparing a 0.5 McFarland Standard suspension by direct colony suspension of* E. coli* ATCC 25922 in saline and diluted 1 : 10 in saline based on the methods described in M100-S23 [18]. The MH plate was inoculated in the same manner of routine disc diffusion. The plate was then allowed to dry for 3 to 10 minutes. Ertapenem disc (10 *μ*g) or Meropenem (10 *μ*g) was then placed on the center of the plate. Three to five colonies were picked by using a loop or a swab of test or QC (ATCC BAA 1706 and ATCC BAA 1705) organism grown overnight on a blood agar plate and was streaked on a straight line out from the edge of the disc. Following 16–24 hours of mesothermic incubation, carbapenemase production is seen with an enhanced growth of* E. coli *wherein carbapenemase produced by the isolate or QC organism ATCC BAA 1705 inactivated either antibiotic Meropenem or Ertapenem that diffused to the media, while negative results showed no enhanced growth of* E. coli*.

### 2.6. Quality Control

Reference strains that were used in this study include* S. aureus* ATCC 25923,* E. coli* ATCC 25922,* E. coli* 35218* P. aeruginosa* ATCC 27853, and* K. pneumoniae* ATCC BAA 1705 and 1706.

## 3. Results

### 3.1. Isolated Pathogens

Out of 130 specimens submitted, 90 specimens were found to be positive in cultures with 96 isolates. Most of the positive samples came from patients aged 60 years old and above encompassing a total of 87.77% and are mostly males (65.55%). Also, 7.79% of the samples came from aged 30–59 years, while 4.44% came from pediatric patients. Most of the samples were from ICU (62.5%), Private Rooms (18.75%), Wards (9.38%), Outpatients (4.93%), and NICU/PICU (4.44%). Based on the microbiological data collected,* P. aeruginosa *was the prevailing bacteria with 41.66%. The number and frequency of other microbes were* K. pneumoniae* (16.67%),* Enterobacter gergoviae* (6.25%), non-*Candida albicans* (7.29%),* Enterobacter aerogenes* and* Burkholderia cepacia* (5.2%),* Enterobacter cloacae* and* Escherichia coli* (4.17%),* A. baumannii* (3.12%),* Candida albicans* and* Citrobacter koseri* (2.08%), and* Serratia marcescens* (1.04%).

### 3.2. Antimicrobial Susceptibility

**Figure 1:**
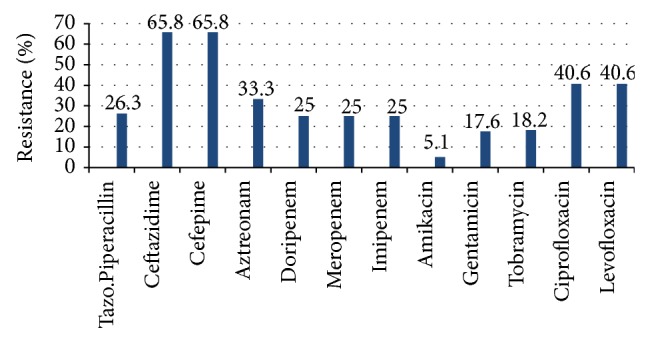
Antibiotic resistance of the isolated* P. aeruginosa* against various antibiotics.

**Figure 2:**
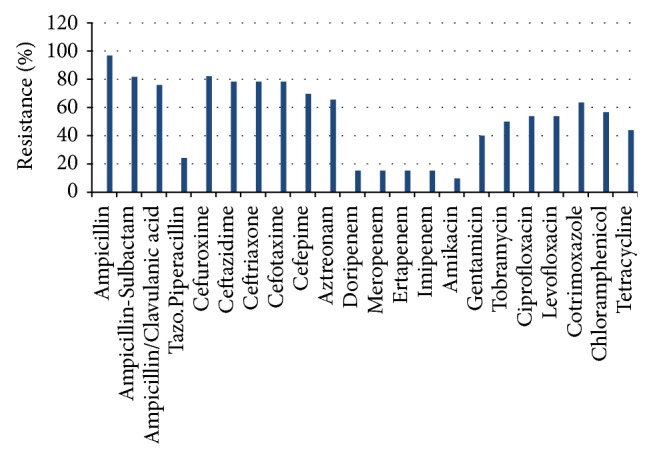
Antibiotic resistance of the isolated Enterobacteriaceae against various antibiotics.

Antimicrobial susceptibility also revealed that* P. aeruginosa *has the highest resistance against Cefepime and Ceftazidime with 65.8% (CI 48.6–79.9%), followed by Ciprofloxacin and Levofloxacin with 40.6% (CI 24.2–59.2), and Amikacin with 5.1% (CI 0.9–18.6) as the lowest, as reflected in Figure 1. On the other hand, Enterobacteriaceae have the most resistance against ampicillin with 96.9% (CI 82.0–99.8%), cefuroxime with 82.1% (CI 62.4–93.2), and ampicillin-sulbactam 81.8% (CI 63.9–92.4) but with least resistance to Amikacin with 9.7% (CI 2.5–26.9%) followed by carbapenems Ertapenem, Imipenem, and Meropenem with 15.2% (CI 5.7–32.7). The data for the susceptibility of Enterobacteriaceae is shown in Figure 2.

Antibiotic susceptibility pattern of* B*.* cepacia*,* A. baumannii*, and* Candida* species was not included due to the fact that they did not meet the minimum number of 30 isolates as per recommendation in the M39-A4 manual [20]. However, it is interesting to note that 2 out of 3 isolates of* A. baumannii* were pan-resistant to all tested antibiotics, 2 out of 9* Candida *species were resistant to fluconazole, and all* B. cepacia* were sensitive to the tested antibiotics. Additionally, among the 40* P. aeruginosa* isolates, 25% were resistant to carbapenems, Imipenem, and Meropenem, yet none of the 25% were positive for metallo-*β*-lactamase production by Imipenem-EDTA synergistic test. Moreover, out of 39* Enterobacteriaceae*, 78.3% were Extended Spectrum *β*-Lactamase (ESBL) producing and 10.26% were carbapenemase producing isolates.

## 4. Discussions

Out of 130 samples submitted for microbial culture 90 (69.23%) were found positive for microbial growth. This finding is most likely due to the reason that endotracheal tubes alter host defenses, impair mechanical clearance, and can cause trauma or inflammation [21]. The majority of the patients subjected to tracheal aspirates are mostly male and age 65 years of age. It was also noted that majority of the patients were admitted in the ICU since these patients were critically ill and have the most chance of acquiring health care acquired infections [11]. Colonization is a matter of concern because admissions on the said units are patients that are critically ill due to their immunological status or age [10]. This finding was also at par with the findings of Khosravi et al. [10].

The isolated bacteria in this study are often the causative agents of lower respiratory health care related infections, as mentioned by Craven and Hjalmarson [7], Resende et al. [5], and Vanhems et al. [6].* P. aeruginosa* is rarely found as a part of the microbial flora of healthy populace, but it was noted for its liking for moist environment like aqueous solutions used in medical care and becomes potentially problematic in the hospital environment [22, 23]. Furthermore,* P. aeruginosa* can also be present in some objects like aerators, sinks, respiratory equipment, and any other water sources [23]. It can also colonize gastrointestinal tract and moist body sites. Together with* Acinetobacter *species, these bacteria can be passed from person to person or environmental contamination [22]. Moreover, bacteria like* K. pneumoniae*,* E. coli*, and* S. marcescens* can be present in urinary catheters, equipment, and other contaminated fluids that can survive in inanimate surfaces for months [23, 24] and can even live longer compared to gram positive bacteria. The above findings are comparable to the study of Khosravi et al. [10] and Resende et al. [5] (2013), but in contrast to the data of Vanhems et al. [6] where* P. aeruginosa* was reported to be infrequent. The study of Jakribettu and Boloor [8] also showed similar series of pathogens but* K. pneumoniae* was found to be the most isolated pathogen (34%) rather than* P. aeruginosa* (20%). As a subject of concern,* P. aeruginosa* and* Acinetobacter *species have higher rate of pulmonary recurrence suggesting that longer courses of therapy are needed [12].

Frequency of multidrug resistance is highly emphasized in this study. Current data reveals that most bacteria such as Enterobacteriaceae and* P. aeruginosa* were least resistant to Amikacin with 9.7% and 5.1%, respectively. Alternately, Enterobacteriaceae had the most resistance to ampicillin followed by cefuroxime which conforms with the data of Jakribettu and Boloor [8], and with Khanal et al. [9]. Even though Amikacin was found to be the antibiotic with the least resistance against Enterobacteriaceae and* P. aeruginosa*, this antibiotic however is known to cause a series of side effects including nephrotoxicity [25], Fanconi-like or Bartter-like syndrome, and hearing loss [26] making any of the carbapenems [27] and Piperacillin/Tazobactam still worth to be one of the most effective drugs for treatment as of this time. As with this study, it was also observed that 78.3% of the isolates were found to be resistant to third generation cephalosporins and 58% to fluoroquinolones, respectively.

The presence of multidrug resistant bacteria specifically ESBL and carbapenemase producing Enterobacteriaceae and carbapenem-resistant* P. aeruginosa* are of great threats noting that carbapenem is still considered as the last resort in treating multidrug resistant [28] bacteria. The number of ESBL confirmed by double disc synergistic test also corresponds to the number of isolates that were resistant to third generation cephalosporins, given that susceptibility to these drugs is also accounted for the screening of ESBL in M100-S23 guidelines of CLSI [18]. It is also worthwhile to mention that these bacteria are capable of causing an outbreak in hospital setting especially in the ICU [22, 28, 29]. These organisms can also persist in the environment for months and can be transmissible as nosocomial pathogens as suggested in the study of Kramer et al. in 2006 [24]. Sydnor and Perl [23] also demonstrated that infections with MDR bacteria like ESBL mean increase in length of hospital stay, increase in hospital charges, and increase in mortality rate. Also included in the list are carbapenemase producing organisms which are considered notorious and alarming for its developing resistance to almost all currently available antibiotics, not mentioning its gene (NDM-1) present on its plasmid that is easily transferrable to other organisms.

Overall, this study presents substantiation and without much difference with other literatures about the microbial content of tracheal aspirates and their resistance to the various antibiotics.

## 5. Conclusions

The data generated by this study conclude that the most common pathogens isolated in tracheal and endotracheal aspirates are* P. aeruginosa* and members of the family Enterobacteriaceae such as* K. pneumoniae*,* E. coli*, and* Enterobacter* spp. which are most susceptible to Amikacin. Production of ESBL among Enterobacteriaceae was evident so as their resistance to carbapenems. Though resistance to carbapenems was 15.2%, the actual carbapenemase producing Enterobacteriaceae from these isolates was 10.26%.
